# Is emotion dysregulation correlated to depressive and manic symptoms of bipolar disorder? Results from a systematic review and network meta-analysis

**DOI:** 10.1192/j.eurpsy.2023.625

**Published:** 2023-07-19

**Authors:** V. Oliva, M. De Prisco, G. Fico, A. Murru, M. Fornaro, A. Serretti, J. Radua, E. Vieta

**Affiliations:** ^1^Bipolar and Depressive Disorders Unit, Institute of Neuroscience, Hospital Clinic, University of Barcelona, IDIBAPS, CIBERSAM, Barcelona, Spain; ^2^Department of Biomedical and Neuromotor Sciences, University of Bologna, Bologna; ^3^Section of Psychiatry, Department of Neuroscience, Reproductive Science and Odontostomatology, Federico II University of Naples, Naples, Italy; ^4^Imaging of Mood- and Anxiety-Related Disorders (IMARD) Group, IDIBAPS, Barcelona, Spain; ^5^Early Psychosis: Interventions and Clinical-Detection Lab, Institute of Psychiatry, Psychology & Neuroscience, King’s College London, London, United Kingdom; ^6^Centre for Psychiatric Research and Education, Department of Clinical Neuroscience, Karolinska Institutet, Stockholm, Sweden; ^7^CIBERSAM, Instituto de Salud Carlos III, Madrid, Spain

## Abstract

**Introduction:**

Emotion dysregulation (ED) is a multidimensional construct involving the lack of awareness, understanding and acceptance of emotions, a reduced access to adaptive and appropriate strategies to modulate the intensity or duration of emotional responses, and the inability to control behaviors in accordance with desired goals when experiencing negative emotions. ED is outlined in the general population and several psychiatric disorders, including bipolar disorder (BD), and influences its clinical course and management, quality of life, and daily social functioning.

**Objectives:**

The objective of this systematic review was to examine the correlations between maladaptive (i.e., positive and negative rumination, negative focus, risk taking behaviors, suppression, and dampening) and adaptive (i.e., cognitive reframing, adaptive coping, and acceptance) strategies of emotion regulation (ER) and depressive and manic symptoms of BD.

**Methods:**

We searched the literature from inception to April 12, 2022, and included studies focusing on ER/ED assessed with a validated scale. We conducted multiple pairwise meta-analyses for correlations between ED dimension (or overall ED) and the measures of depressive and manic symptoms of BD, and separate Bayesian network meta-analyses to examine which aspects of emotion regulation were most closely associated with depressive and manic symptoms of BD. The Pearson’s r coefficients were adjusted using sample-size weights and Fisher’s r-to-z transformed was conducted.

**Results:**

A total of 13,826 records was identified and, after duplicate removal and title/abstract evaluation, 442 were explored at the full text. Sixteen studies were finally included. Results from pairwise meta-analyses are shown in Figure 1, results from network meta-analyses in Figure 2 and 3. Both depressive and manic BD symptomatology were found to be related to maladaptive ER strategies, with the only difference of *positive rumination,* associated only to manic symptoms. *Negative rumination* and *risk-taking* behaviors were the strategies more correlated to both manic and depressive symptoms, as confirmed by both pairwise metanalyses and network metanalyses. On the other hand, depressive symptomatology appeared more correlated with decreased adaptive strategies than manic symptomatology.

**Image:**

**Figure:**
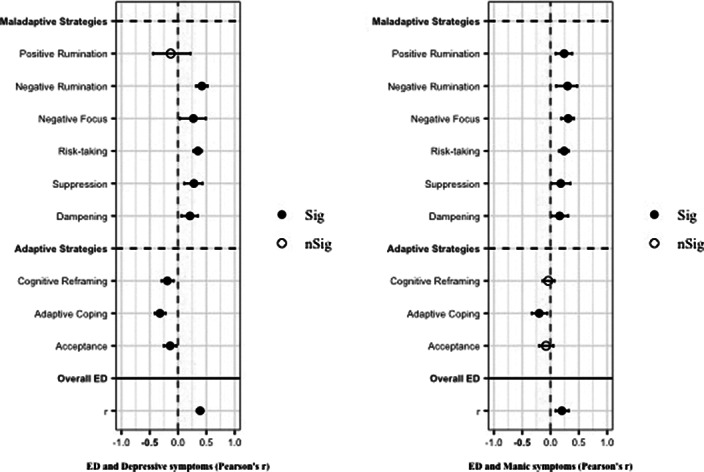

**Image 2:**

**Figure:**
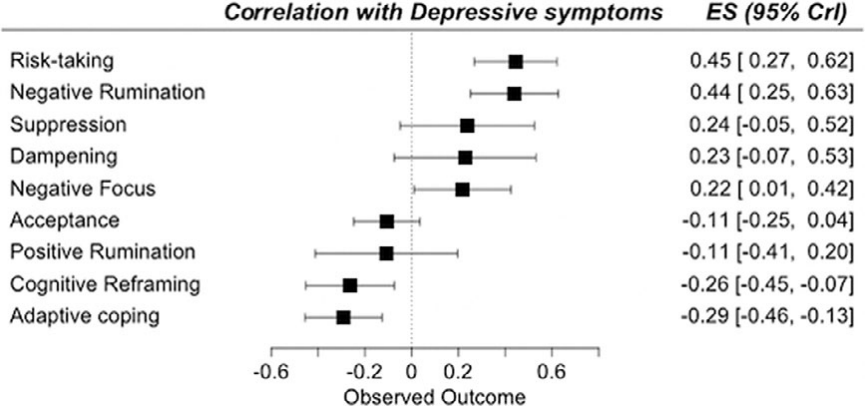

**Image 3:**

**Figure:**
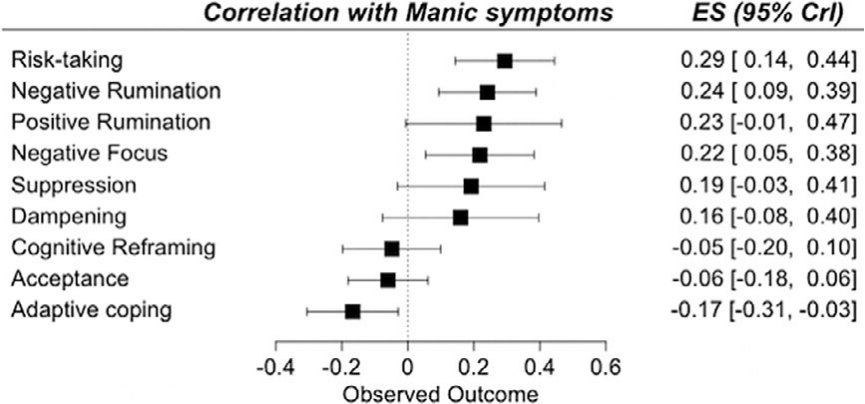

**Conclusions:**

ED has a significant correlation with BD symptomatology, therefore it should be explicitly considered during clinical assessment, diagnosis, and intervention on BD, and specific treatments should be implemented. More studies, and with longitudinal design, are needed to better explore these associations and their causal direction. In addition, future studies should mainly focus on the complex interactions between cognitive, social, and cultural aspects, and biological correlates to improve knowledge on a topic that is still poorly investigated.

**Disclosure of Interest:**

V. Oliva: None Declared, M. De Prisco: None Declared, G. Fico Grant / Research support from: “La Caixa” Foundation (ID 100010434 - fellowship code LCF/BQ/DR21/11880019), Consultant of: Angelini, Janssen-Cilag and Lundbeck, A. Murru Grant / Research support from: Spanish Ministry of Science and Innovation (PI19/00672) integrated into the Plan Nacional de I+D+I and co-financed by the ISCIII-Subdirección General de Evaluación and the Fondo Europeo de Desarrollo Regional (FEDER), Consultant of: Angelini, Idorsia, Lundbeck, Pfizer, Takeda, M. Fornaro: None Declared, A. Serretti Consultant of: Abbott, Abbvie, Angelini, AstraZeneca, Clinical Data, Boehringer, Bristol-Myers Squibb, Eli Lilly, GlaxoSmithKline, Innovapharma, Italfarmaco, Janssen, Lundbeck, Naurex, Pfizer, Polifarma, Sanofi, Servier, and Taliaz, J. Radua Grant / Research support from: Spanish Ministry of Science and Innovation (PI19/00394, CPII19/00009) integrated into the Plan Nacional de I+D+I and co-financed by the ISCIII-Subdirección General de Evaluación and the Fondo Europeo de Desarrollo Regional (FEDER) and the Instituto de Salud Carlos III, E. Vieta Grant / Research support from: Spanish Ministry of Science and Innovation (PI18/00805, PI21/00787) integrated into the Plan Nacional de I+D+I and co-financed by the ISCIII-Subdirección General de Evaluación and the Fondo Europeo de Desarrollo Regional (FEDER); the Instituto de Salud Carlos III; the CIBER of Mental Health (CIBERSAM); the Secretaria d’Universitats i Recerca del Departament d’Economia i Coneixement (2017 SGR 1365), the CERCA Programme, and the Departament de Salut de la Generalitat de Catalunya for the PERIS grant SLT006/17/00357. Thanks the support of the European Union Horizon 2020 research and innovation program (EU.3.1.1. Understanding health, wellbeing and disease: Grant No 754907 and EU.3.1.3. Treating and managing disease: Grant No 945151), Consultant of: AB-Biotics, AbbVie, Angelini, Biogen, Boehringer-Ingelheim, Celon Pharma, Dainippon Sumitomo Pharma, Ethypharm, Ferrer, Gedeon Richter, GH Research, Glaxo-Smith Kline, Janssen, Lundbeck, Medincell, Novartis, Orion Corporation, Organon, Otsuka, Rovi, Sage, Sanofi-Aventis, Sunovion, Takeda, and Viatris

